# ER stress activation in the intestinal mucosa but not in mesenteric adipose tissue is associated with inflammation in Crohn’s disease patients

**DOI:** 10.1371/journal.pone.0223105

**Published:** 2019-09-26

**Authors:** Andressa Coope, Lívia Bitencourt Pascoal, José Diego Botezelli, Francesca Aparecida Ramos da Silva, Maria de Lourdes Setsuko Ayrizono, Bruno Lima Rodrigues, Marciane Milanski, Rita Barbosa Carvalho, João José Fagundes, Lício Augusto Velloso, Raquel Franco Leal

**Affiliations:** 1 IBD Research Laboratory, Colorectal Surgery Unit, School of Medical Sciences, University of Campinas (UNICAMP), Campinas, São Paulo, Brazil; 2 Laboratory of Metabolic Disorders, School of Applied Sciences, University of Campinas (UNICAMP), Limeira, São Paulo, Brazil; 3 Department of Pathology, Gastrocenter, School of Medical Sciences, University of Campinas (UNICAMP), Campinas, São Paulo, Brazil; 4 Laboratory of Cell Signaling, School of Medical Sciences, University of Campinas (UNICAMP), Campinas, São Paulo, Brazil; Wayne State University, UNITED STATES

## Abstract

Chronic/abnormal activation of endoplasmic reticulum (ER) stress is linked to the exacerbation of the inflammatory process and has been recently linked to Crohn’s disease (CD) pathophysiology. We investigated the intestinal mucosa and the mesenteric adipose tissue (MAT) collected from CD patients with active disease (CD group) and from non-IBD patients (CTR group) to study ER stress activation and to address tissue-specific modulation in CD. The intestinal mucosa of CD patients showed an upregulation in the expression of ER stress related genes, including *ATF3*, *DNAJC3*, *STC2*, *DDIT3*, *CALR*, *HSPA5* and *HSP90B1*. Results showed that *EIF2AK3* gene was upregulated, along with increased protein expression of p-eIF2α and p-eIF2α/eIF2α ratio. Additionally, *ERN1* gene expression was upregulated, along with an increased spliced/activated form sXBP1 protein. Despite the upregulation of *ATF6* gene expression in the intestinal mucosa of CD patients, no differences were found in ATF6 protein expression. Lastly, the analysis of MAT revealed unchanged levels of ER stress markers along with no differences in the activation of UPR. However, chaperone gene expression was modulated in the MAT of CD patients. To conclude, our results address tissue-specific differences in UPR activation in CD and point the ER stress as an important pro-inflammatory mechanism in CD, specifically in the intestinal mucosa.

## Introduction

Crohn’s Disease (CD) is a chronic multifactorial inflammatory bowel disease (IBD) characterized by transmural granulomatous inflammation, mucosal immune cell activation, abnormal immune response and cytokine imbalance, due to a complex interaction encompassing genetic predisposition, environmental risk factor, microbiota dysregulation and impaired mucosal immune system [1,2]. The inflammatory transcriptional profile in CD is different and complex throughout the tissues. A study conducted by our group demonstrated for the first time that the mesenteric adipose tissue (MAT) of CD patients shows an increased activation of the STAT1 (Signal transducer and activator of transcription 1) pathway, along with an increase of the anti-inflammatory IL-10 (Interleukin-10) cytokine, and decreased activation of IκB (inhibitor of kappa B). Additionally, reinforces the predominance of the pro-inflammatory NF-κB (nuclear factor kappa B) activation in the intestinal mucosa with increased TNF-α (Tumor necrosis factor-α), IL-1β (Interleukin-1β) and IL-23 (Interleukin-23) activation [3]. However, the MAT of CD patients shows an impaired autophagy and apoptosis regulation [4,5], which display the complex interplay between intestinal mucosa and MAT in the pathogenesis of CD and highlights the importance of dissecting the molecular mechanisms that drive inflammation.

Recent studies have implicated the activation of ER stress in the pathogenesis of IBD, as it contributes to the perpetuation of the inflammatory process and autoimmunity [6,7]. Aberrant/inadequate UPR response contributes to the development of autoimmune diseases by creating disturbances in immune and non-immune cells [7]. The ER is an important organelle in eukaryotic cells responsible for biosynthesis, folding, assembly and modification of membrane and secretory proteins. Nearly one-third of all proteins are processed in the ER. A number of stressor factors, like microbial pathogens, radiation, hypoxia, nutrient deprivation, toxins, among other environmental stressors, can cause accumulation of misfolded or unfolded proteins in the ER during protein synthesis, leading to ER stress. Interestingly, eukaryotic cells have evolved a mechanism to cope with the increased protein cargo, to ensure the fidelity of protein folding, and to reach homeostasis known as the unfolded-protein response (UPR) [8]. Three UPR axis are activated in response to ER stress: IRE1/sXBP1 (inositol requiring enzyme 1/spliced form of X-box binding protein-1), ATF6 (activating transcription factor 6) and PERK/eIF2α (PKR-like eukaryotic initiation factor 2α kinase/ eukaryotic translation initiation factor 2 α). Together, they coordinate a response to the unfolded/misfolded proteins accumulation.

Other key components of the UPR response are the chaperones, such as 78-glucose regulated protein (GRP78/BiP) [8,9] and 94-glucose regulated protein (GRP94, gp96, endoplasmin, Erp99, HSP108 or Tra-1) [10,11]. Normal UPR response is important for the development, survival and function of secretory cells like immunoglobulin-producing plasma cells, survival and function of dendritic cells and macrophages, in addition to maintenance of normal epithelial function [6,7].

The host’s ability to deal with the ER stress is possibly involved in the pathogenesis of intestinal inflammation. ER stress-induced inflammation is an important protective response for the organism’s function and survival. However, it becomes detrimental when chronically engaged [8,12,13]. The UPR is needed to activate the inflammatory response through a variety of inflammatory signaling pathways including IκB kinase (IKK)/NF-κB and c-Jun N-terminal Kinase (JNK)/AP1. IRE1α activates JNK/AP1 pathway triggering upregulation of inflammatory genes [14]. Interestingly, IRE1α, PERK and ATF6 activates IKK/NF-κB through multiple and distinct mechanisms [15–18]. Chronic UPR activation leads to exacerbation of the inflammatory process and can ultimately result in cellular apoptosis triggering cellular and tissue damage [8]. Thus, abnormal/chronic ER stress activation can contribute to the upregulation of intestinal inflammatory pathways observed in CD patients [6,12,13]. Therefore, evaluating the transcriptional and protein levels of UPR components is an accurate way of studying ER stress, as well as detecting whether chronic damage is already occurring as a result of increased apoptosis pathway activation [8,19,20].

In this study, we investigated the activation of the three UPR branches in the intestinal mucosa and in the MAT of CD and control patients, and showed for the first time in the literature, the tissue-specific differences of ER stress activation in CD patients, which is important for addressing tissue-specific drug response and new drug development in the field.

## Material and methods

### Patients and ethics statement

Samples from intestinal mucosa and MAT, located near the affected intestinal area, were taken from patients with ileocecal CD (CD Group) after surgical resection. The control groups were composed of patients who underwent intestinal resection for non-inflammatory disease, with normal distal ileum (MAT biopsies near the ileum were collected during the surgery—CTR group of MAT) and patients with normal ileocolonoscopy (mucosa biopsies were taken—CTR group of intestinal mucosa). The intestinal mucosa control group was composed by individuals who underwent colonoscopy for surveillance of colorectal cancer or investigation of bleeding, without macroscopic abnormalities after examination. Afterwards, histological analysis (Haematoxylin & Eosin, H&E) was performed to confirm the normal findings of the colonoscopy.

Concerning the CD group, disease activity was assessed by colonoscopy before surgery and all patients had more than 250 points in the Crohn’s disease activity index (CDAI). Table 1 shows the clinical and demographic characteristics of patients included in the study. We selected CD patients with distal ileal involvement because this is the most common anatomical site of disease affection. All patients included in the study had an albumin level higher than 3mg/dL prior to the surgical procedure, which is important to decrease the complication rates after surgery. Malnourished patients received nutritional support before surgery. Sample collection was performed when all patients were nourished and had a reasonable nutritional condition. Body mass index (BMI) was below 25, which excluded obesity as a risk factor. Moreover, the CD patients fasted prior to surgery (12 hours), as did the control subjects who underwent colonoscopy, thus excluding this variable from the study.

**Table 1:** Clinical and demographic characteristics of the patients included in the study for the qRT-PCR analysis.

	**Patients included in the qPCR analysis**		
	**CD Group**	**Control Group** **(intestinal mucosa)**	**Control Group** **(MAT)**
**Number**	10	8	8
**Gender (M/F)**	4/6	3/5	5/3
**Age (years)**	32.5 [20–66]	56 [33–66]	54.5 [39–67]
**Disease duration (years)**	6 [1–20]	-	-
Age at diagnosis (A1/A2/A3)*	1/7/2	-	-
Location (L1/L2/L3/L4)*	4/0/6/0	-	-
Behaviour (B1/B2/B3)*	0/4/6	-	-
**Perianal disease (yes/no)**	2/8	-	-
**Immunosuppressant (yes/no)**	7/3	-	-
**Anti-TNFα (yes/no)**	10/0	-	-
**CDAI**	321 [261.4–609.6]	-	-
Presence of ulcers (yes/no)**	10/0	0/10	0/10
	**Patients included in the Western Blot analysis**		
	**CD Group**	**Control Group** **(intestinal mucosa)**	**-**
**Number**	19	7	-
**Gender (M/F)**	7/12	2/5	-
**Age (years)**	37 [20–70]	56 [44–69]	-
**Disease duration (years)**	6 [1–30]	-	-
Age at diagnosis (A1/A2/A3)*	1/15/3	-	-
Location (L1/L2/L3/L4)*	6/0/13/0	-	-
Behaviour (B1/B2/B3)*	0/11/8	-	-
**Perianal disease (yes/no)**	2/17	-	-
**Immunosuppressant (yes/no)**	7/12	-	-
**Anti-TNFα (yes/no)**	14/5	-	-
**CDAI**	302.5 [162–428]	-	-
Presence of ulcers (yes/no)**	19/0	0/7	-

Numerical variables are described as median [min, max] and categorical variables as absolute frequencies.

*Montreal Classification.

**Presence of ulcers in the ileum evaluated by colonoscopy examination.

CD = Crohn’s disease. MAT = mesenteric adipose tissue. M = male. F = female. TNF = tumor necrosis factor. CDAI = Crohn’s disease Activity Index.

In this exploratory observational study, a formal sample size calculation was not performed. The study was conducted in accordance with the Declaration of Helsinki and approved by the Ethics Committee of the University of Campinas (n° 356/2009 and CAAE n° 0271.0.146.000–09). All participants read and signed a written informed consent form for study participation. The study was carried out at the IBD Research Laboratory and the Laboratory of Cell Signaling of the School of Medical Sciences, University of Campinas (UNICAMP).

### Western blot analysis

For total protein extract preparation, the fragments of intestinal and MAT were previously snap-frozen and stored at—80°C. They were homogenized in approximately 10 volumes of solubilization buffer containing 1% Triton X-100, 100 mM Tris (pH 7.4), 100 mM sodium pyrophosphate, 100 mM sodium fluoride, 10 mM EDTA, 10 mM sodium orthovanadate, 2 mM PMSF (phenylmethylsulfonyl fluoride) and 0.1 mg/mL aprotinin at 4°C in a “Polytron PTA 20S Generator” (model PT 10/35; Brinkmann Instruments, Westbury, NY). Insoluble material was removed by centrifugation (40 min at 12000 rpm at 4°C). Protein concentrations were determined using the Pierce^™^ BCA Protein Assay Kit (Thermo, NY, USA). A 50 μg aliquots were separated by SDS-PAGE, transferred to nitrocellulose membranes, and blotted with specific antibodies. Reagents for SDS-PAGE and immunoblotting were from BioRad Laboratories (Richmond, CA). Phenylmethylsulfonyl fluoride, aprotinin, Triton X-100, Tween 20, and glycerol were from Sigma (St. Louis, MO). Nitrocellulose paper (BA85, 0.2 mm) came from Amersham (Aylesbury, UK). Molecular weights of proteins were assessed using the PageRulerTM from Fermentas (Glenburnie, MD). Ponceau S staining was applied to determine the loading control and protein transfer efficiency as described by Fortes et al [21] and by Romero-Calvo et al [22]. In summary, immediately after the wet transfer, membranes were washed for five minutes in deionized water to remove the transfer buffer. The membranes were washed in Ponceau S staining (5%) for 5 minutes. The solution was removed, and membranes were washed for 5 minutes in deionized water to remove the background. After this process, the membranes were scanned and converted to black and white images. Using optical densitometry (UN-SCAN-IT gel), lanes were selected and the total pixels in each lane was used as loading control (S1 Fig). The electrophoretic gels and blots, and the compliance with the digital images are shown in S1 Appendix.

The antibodies, anti-eIF2α (sc11386, rabbit polyclonal) was purchased from Santa Cruz Biotechnology (CA, USA) and anti-phosphor-[Ser51] eIF2α (ab32157, rabbit monoclonal) were purchased from Abcam; anti-ATF6 (Fulllength and Active/Cleaved Forms. Clone 70B1413.1 IMG-273, mouse monoclonal) was purchased from Imgenex and purified anti-Xbp-1 (COOH terminus) (619502, rabbit polyclonal) was purchased from BioLegend. The signal was detected by a chemiluminescent reaction (SuperSignal West Pico Chemiluminescent Substrate from Pierce Biothecnology, Inc. Rockford, IL) and quantified by optical densitometry (UN-SCAN-IT gel).

### RNA extraction and cDNA synthesis

Total RNA was extracted using the RNeasy Mini Kit (Qiagen—Cat No./ID: 74104), according to the manufacturer’s instructions. RNA purity and concentration were determined by UV spectrophotometry at 260 nm. For cDNA synthesis, we used the High Capacity cDNA Reverse Transcription Kit (Applied Biosystems, Foster City, CA, USA), according to the manufacturer’s instructions. The cDNA was diluted to the concentration required for the efficient amplification of each gene.

### Quantitative real-time PCR (qPCR)

Real-time quantitative PCR reactions were performed using the TaqMan ^™^ system (Applied Biosystems). Taqman Primers used was: EIF2AK3 (Hs_00178128_m1), ATF3 (Hs_00231069_m1), ATF6 (Hs_00232586_m1), calreticulin (Hs_00189032_m1), STC2 (Hs_00175027_m1), DNAJC3 (Hs_00534483_m1), DDIT3 (Hs_00358796_g1), ERN1 (Hs_00980095_m1), HSPA5 (Hs_99999174_m1), HSP90B1 (Hs_00427665_g1), and glyceraldehyde-3-phosphate dehydrogenase (GAPDH) (4326317E) were obtained from Applied Biosystems. The Taqman primer: DEFA5A (NM_021010) was obtained from Integrated DNA Technologies (IDT). All measurements were normalized by the expression of GAPDH gene, which is generally considered a stable housekeeping gene. Life Technologies follows the pattern of assigning FAM^™^ dye as the reporter for the target assay and assigning VIC® dye as the reporter for the normalizer assay. RT-PCR was performed on resulting cDNA, using the manufacturer’s protocol. RT-PCR amplification consisted of an initial denaturation step 45 cycles of denaturation (95°C for 3 min), annealing (95°C for 5 s) and extension (60°C for 30 s), followed by a final incubation at 60°C for 1 minute. Each PCR contained 40 ng of reverse-transcribed RNA, 2.5 μl of each specific primer, Taqman Universal master mix (4369016, Life Technologies) and RNAse-free water at a final volume of 10 μl. Real-time PCR analysis of gene expression was carried out on the Applied Biosystem StepOne^TM^ detection system. Gene expression was determined using the delta-delta Ct method.

### Hematoxylin and eosin (H&E) staining

Paraffin-embedded ileal and MAT blocks of 10 CD patients and 8 controls were cut at 5 μm and used for histological analysis. Sections were stained with H&E dye. Photomicrographs were recorded using the *Leica DM4500* microscope and the *Leica DFC290* digital camera for microscopy with control software (Leica Microsystems, Wetzlar).

### PAS-Alcian blue staining

Paraffin-embedded ileal blocks of one CD patient and one control were cut at 5 μm and used for histological analysis. Sections were stained with PAS-Alcian Blue dye. Photomicrographs were recorded using the *Leica DM4500* microscope and the *Leica DFC290* digital camera for microscopy with control software (Leica Microsystems, Wetzlar).

### Immunohistochemistry (IHC)

Paraffin-embedded ileal blocks of two patients from each group (CTR and CD groups) were cut at 5 μm and used to investigate the localization of specific proteins. The slides preparation included deparaffinization, hydration, followed by antigen retrieval and staining. Antigen retrieval was performed using a citrate buffer solution (10 mM, pH 6.0) for 30 minutes at 95°C. The endogenous peroxidase was blocked performing 3 washes of 10 minutes each using a blocking solution based on hydrogen peroxide (3% H_2_O_2_ 10 vol), followed by washes in distilled water and phosphate buffered saline (PBS, 10 mM, pH 7.4). The primary antibodies were diluted in predetermined titter in a 1% bovine serum albumin (BSA) in PBS buffer and incubated overnight at 4°C. The antibodies employed were: GRP78 (sc13968, rabbit polyclonal), GRP94 (sc11402, rabbit polyclonal) purchased from Santa Cruz Biotechnology; anti-phosphor-[Ser51] eIF2α (ab32157, rabbit monoclonal) purchased from Abcam; purified anti-Xbp-1 (COOH terminus) (619502, rabbit polyclonal) purchased from BioLegend; PE Mouse Anti-Human CD45 (555483) purchased from BD Pharmingen; and Lysozyme (RB-372-A1, rabbit polyclonal) purchased from Thermo Scientific. For detection we washed the slides with PBS and incubated for 30 minutes with Advance^TM^ HRP Link (DAKO, K4068) followed by washes in PBS buffer and incubation with Advance HRP Enzyme for 30 min. The slides were washed in PBS and incubated with DAB solution (1 drop of the DAB Chromogen per 1 mL of Substrate Buffer) (DAKO, K3468). The slides were rinsed with distilled water and counterstained with Mayer’s Hematoxylin, followed by immersion on 0.037 mol/L ammonia solution. The slides were washed, dehydrated and mounted with Entellan® New (Cat. no. 107961—Merck, Germany). Photomicrographs were recorded using the *Leica DM4500* microscope and the *Leica DFC290* digital camera for microscopy with control software (Leica Microsystems, Wetzlar).

### Statistical analysis

All results are reported as median with interquartile range. Test for distributional adequacy, the *Kolmogorov*-*Smirnov test* was used to investigate if the data follow normal distribution or *Gaussian distribution* (p>0.1). Data were analyzed using the non-parametric Mann-Whitney Test. The level of significance was set at p<0.05. (S1 Table)

### Ethics approval and consent to participate

This study was approved by the Ethics Committee of the University of Campinas (UNICAMP), all patients signed the informed consent form, and were performed in accordance with the Declaration of Helsinki.

## Results

### IRE1/sXBP-1 pathway is activated in the intestinal mucosa, but not in MAT of Crohn’s disease patients

We started this investigation by analyzing the inflammatory status and morphometric parameters of the intestinal mucosa and MAT in CD patients and controls. Histological analysis using H&E staining showed crypt distortion and thickening of the bowel associated with inflammation in the intestinal mucosa of CD patients, compared to the control group. H&E staining in MAT of CD patients showed a reduced adipocyte area and perimeter compared to the control group (S2A Fig). The CD45 staining by immunohistochemistry revealed infiltrating immune cells in the intestinal mucosa and MAT from CD and control groups (S2B Fig).

**Fig 1:**
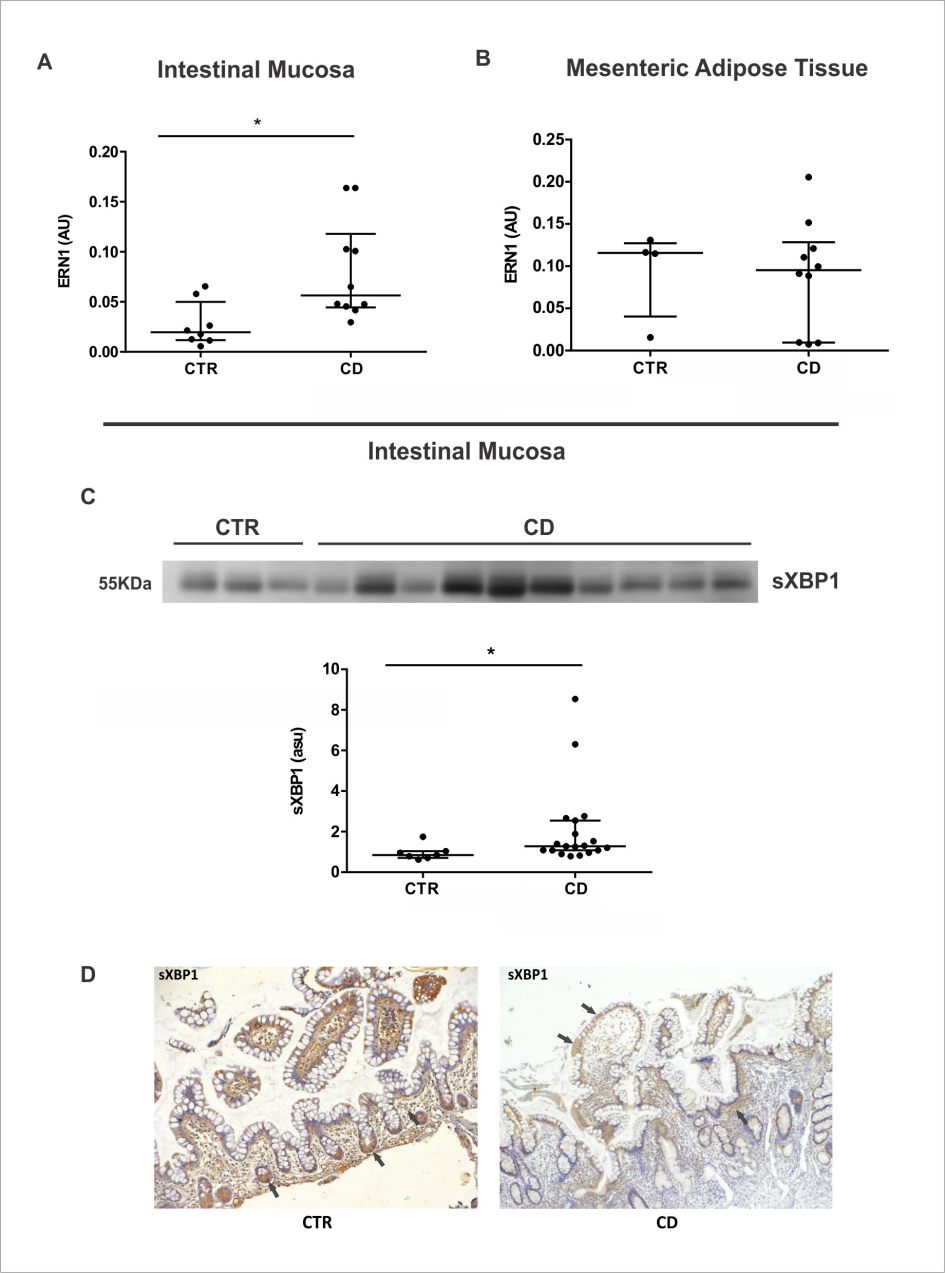
Activation of IRE1/sXBP1 pathway in the intestinal mucosa and in the mesenteric adipose tissue (MAT) of Crohn’s disease patients. mRNA levels (qRT-PCR) of *ERN1* gene was investigated in the intestinal mucosa (A) and in MAT (B) of CD patients (CD group) compared to control group (CTR group).*P < 0.05 and **p < 0.01 are considered statistically significant versus control group. Western blot analysis of the spliced/activated form of XBP1 was evaluated in the intestinal mucosa of CD patients (CD Group) compared to controls (CTR Group) (C). Each band represents one patient. *P < 0.05 and **p < 0.01 are considered statistically significant versus control group. Immunohistochemical analysis of sXBP1 was performed on paraffin-embedded slides from intestinal mucosa of CD and CTR group. The arrows show labeled epithelial cells by immunohistochemistry. Original magnification 100X (D). AU: arbitrary unit. ASU: arbitrary scanning unit.

To better evaluate the ER stress activation, we dissected the UPR branches by qPCR, immunoblotting and immunohistochemistry in CD and CTR samples. IRE1/sXBP1 was the first UPR axis investigated. *ERN1*, which encodes IRE1 protein, is upregulated in the intestinal mucosa (Fig 1A), but is not modulated in the MAT of CD patients compared to respective controls (Fig 1B). Since the measurement of sXBP1 is an accurate form to infer the activation of IRE1 pathway, we investigated the protein expression of sXBP1 using an antibody that specifically recognizes the spliced variant of XBP1. The increased gene expression of *ERN1* is in line with the increased protein expression of the active sXBP1 in the intestinal mucosa of CD patients (Fig 1C), which implies the activation of this UPR branch. The localization of sXBP1 in the intestinal mucosa was investigated by immunohistochemistry. The immunoreactivity of sXBP1 protein is present in the apex of intestinal villi of the CD group, while it was evident in the intestinal crypt base in the control group. Immunostaining also revealed sXBP1 expression in the immune cells in the lamina propria in both groups (Fig 1D).

### ATF6 pathway is not activated in the intestinal mucosa and MAT of Crohn’s disease patients

**Fig 2:**
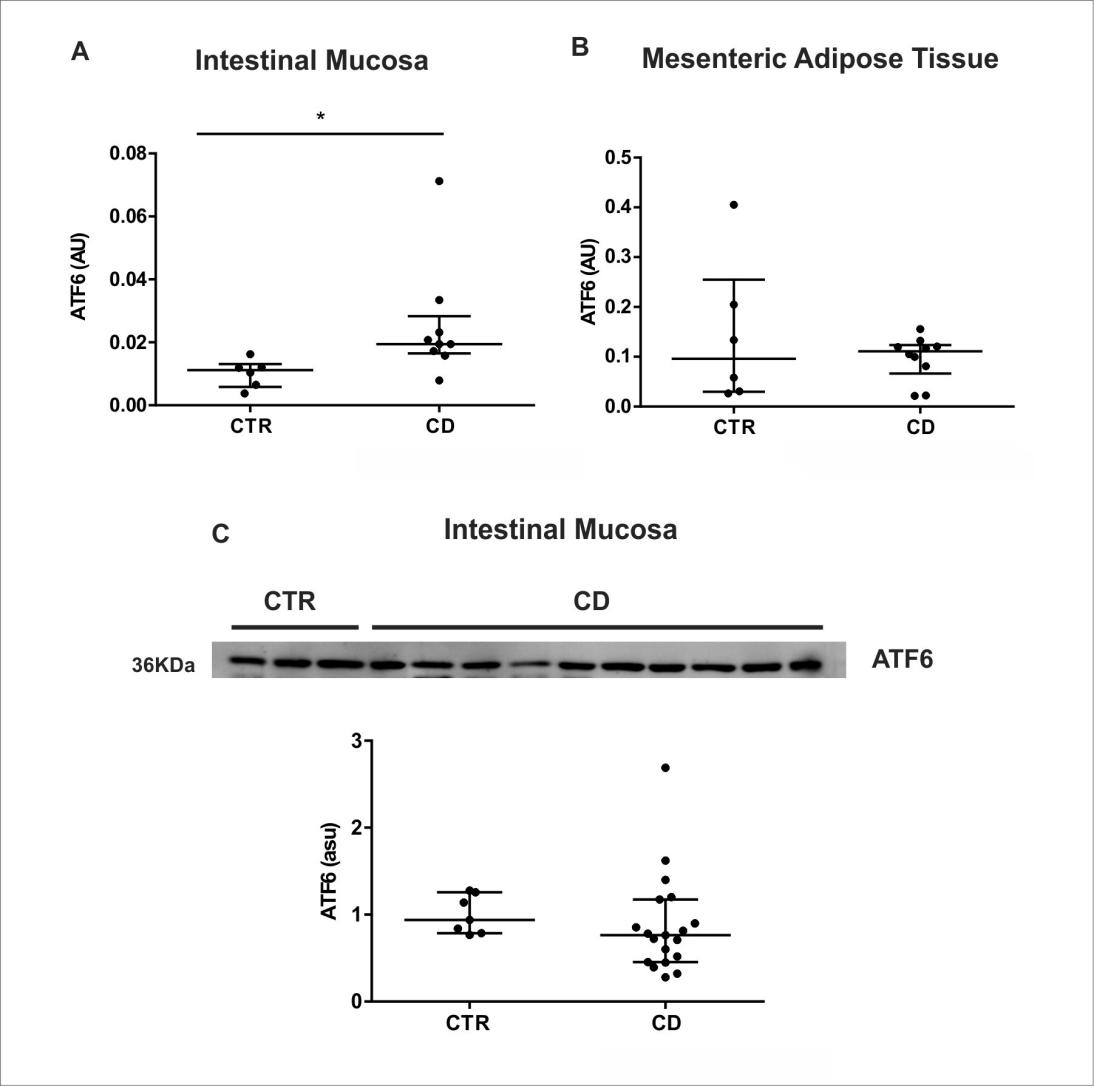
Activation of ATF6 pathway in the intestinal mucosa and in the mesenteric adipose tissue (MAT) of Crohn’s disease patients. mRNA levels (qRT-PCR) of *ATF6* gene was investigated in the intestinal mucosa (A) and in MAT (B) of CD patients (CD group) compared to control group (CTR group). Western blot analysis of the cleaved form of ATF6 protein was evaluated in the intestinal mucosa of CD patients (CD Group) compared to controls (CTR Group) (C). Each band represents one patient. *p < 0.05 and **p < 0.01 are considered statistically significant versus control group. AU: arbitrary unit. ASU: arbitrary scanning unit.

The second UPR axis investigated was the ATF6 pathway. We observed an upregulation of *ATF6* transcriptional levels in the intestinal mucosa of CD patients (Fig 2A). Furthermore, it remained unchanged in the MAT when compared to respective controls (Fig 2B). Despite the changes in *ATF6* gene expression in intestinal mucosa, no differences were found in ATF6 protein levels in both groups (Fig 2C).

### PERK/eif2α pathway is activated in the intestinal mucosa, but not in MAT of Crohn’s disease patients

**Fig 3:**
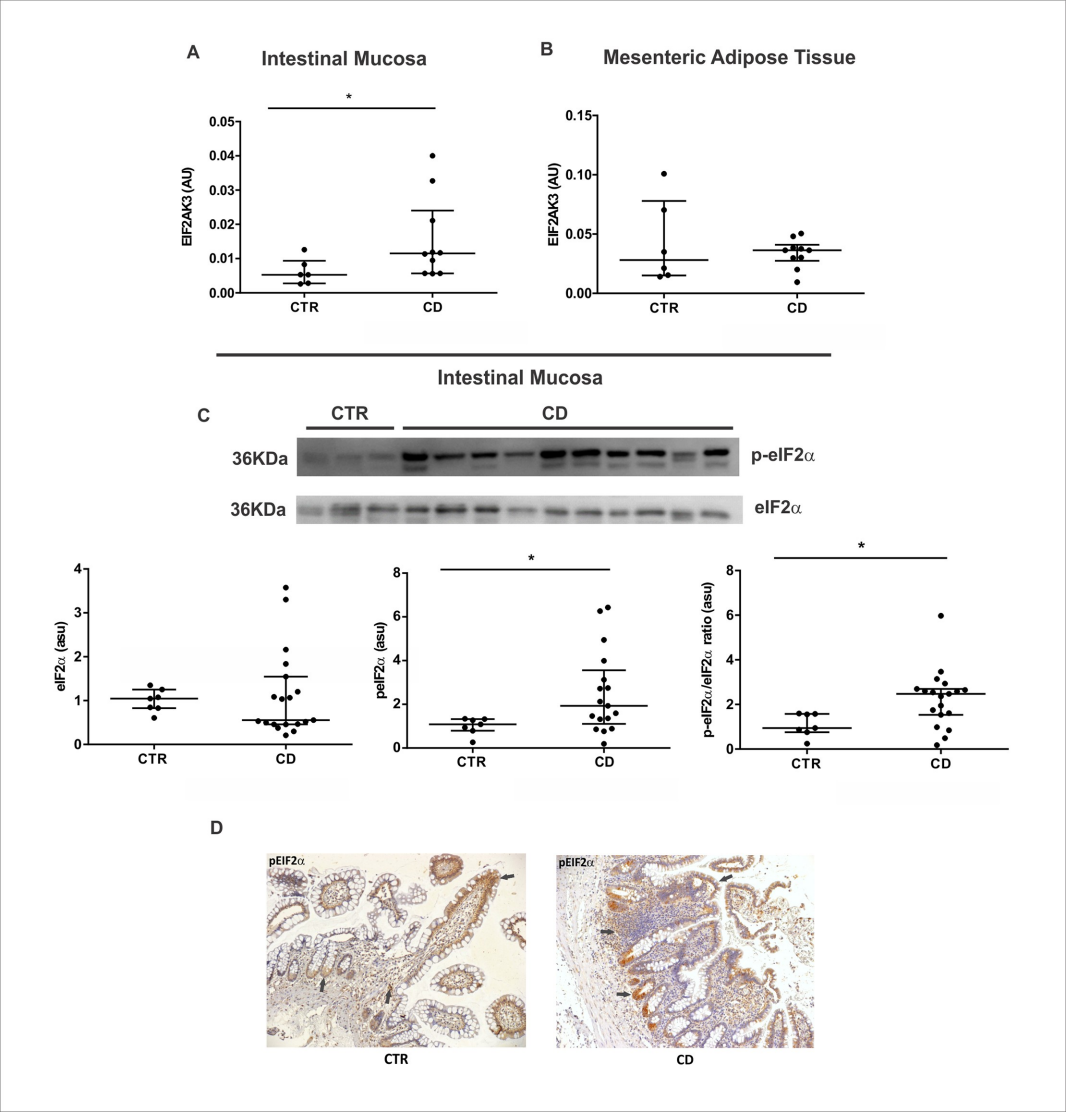
Activation of PERK/eIF2α pathway in the intestinal mucosa and in the mesenteric adipose tissue (MAT) of Crohn’s disease patients. mRNA levels (qRT-PCR) of *EIF2AK3* gene was investigated in the intestinal mucosa (A) and in MAT (B) of CD patients (CD group) compared to control group (CTR group). *p < 0.05 and **p < 0.01 are considered statistically significant versus control group. Western blot analysis of phosphorylated and total form of the protein eIF2α (C), was evaluated in the intestinal mucosa of CD patients (CD Group) compared to controls (CTR Group). Each band represents one patient. *p < 0.05 and **p < 0.01 are considered statistically significant versus control group. Immunohistochemical analysis of p-eIF2α was performed on paraffin-embedded slides from intestinal mucosa of CD and CTR group. The arrows show labeled epithelial cells by immunohistochemistry. Original magnification 100X (D). AU: arbitrary unit. ASU: arbitrary scanning unit.

The third UPR axis investigated was the PERK/eIF2α pathway. The *EIF2AK3*, that encodes the PERK protein, showed an upregulation in the intestinal mucosa (Fig 3A) of CD patients. Conversely, no differences were found in the MAT compared to the respective controls (Fig 3B). The increased expression of PERK in intestinal mucosa corroborates a significant increase of both the p-eIF2α protein levels and the p-eIF2α/eIF2α ratio (Fig 3C). Therefore, we observed the activation of PERK pathway in the intestinal mucosa of CD patients. Additionally, we investigated the location of p-eIF2α in the intestinal mucosa of CD patients by immunohistochemistry and verified the p-eIF2α immunoreactivity in the base of intestinal crypts, in the apex of intestinal villi and in the immune cells from the lamina propria in both groups (Fig 3D).

### Transcriptional evaluation of genes responsive to the UPR pathways implies the ER stress activation in intestinal mucosa, but not in MAT of Crohn’s disease patients

**Fig 4:**
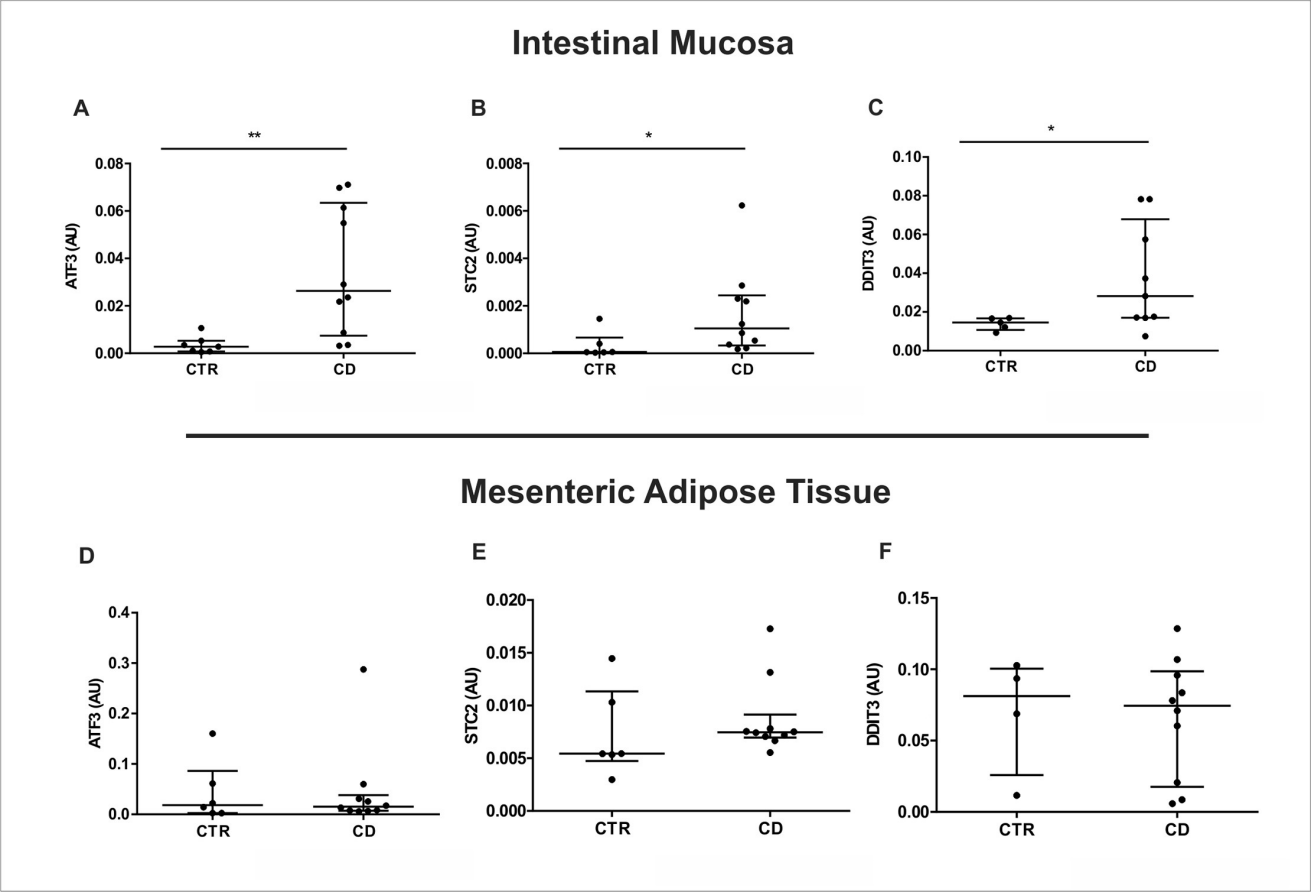
Activation of ER stress responsive genes in the intestinal mucosa and mesenteric adipose tissue (MAT) of Crohn’s disease patients. mRNA levels (qRT-PCR) of *ATF3* (A), *STC2* (B) and *DDIT3* (C) were investigated in intestinal mucosa of CD patients (CD Group) compared to controls (CTR Group). mRNA levels (qRT-PCR) of *ATF3* (D), *STC2* (E) and *DDIT3* (F) were evaluated in MAT of CD patients (CD Group) compared to controls (CTR Group). *p < 0.05 and **p < 0.01 are considered statistically significant versus control group. AU: arbitrary unit.

We evaluated the transcriptional levels of several ER stress responsive genes in biopsies of two different tissues: intestinal mucosa and MAT of CD patients and respective controls. We demonstrate an upregulation of the activating transcription factor 3 (ATF3) (Fig 4A), the calcium-regulatory protein stanniocalcin-2 (Fig 4B), and the DDIT3 (CHOP) (Fig 4C) in the intestinal mucosa of CD patients by Real-Time PCR. Additionally, the transcriptional levels of *ATF3*, stanniocalcin-2 and *DDIT3* remained unchanged in the MAT of CD patients (Fig 4D, 4E and 4F respectively).

### Chaperones modulation in the intestinal mucosa and MAT of Crohn’s disease patients

**Fig 5:**
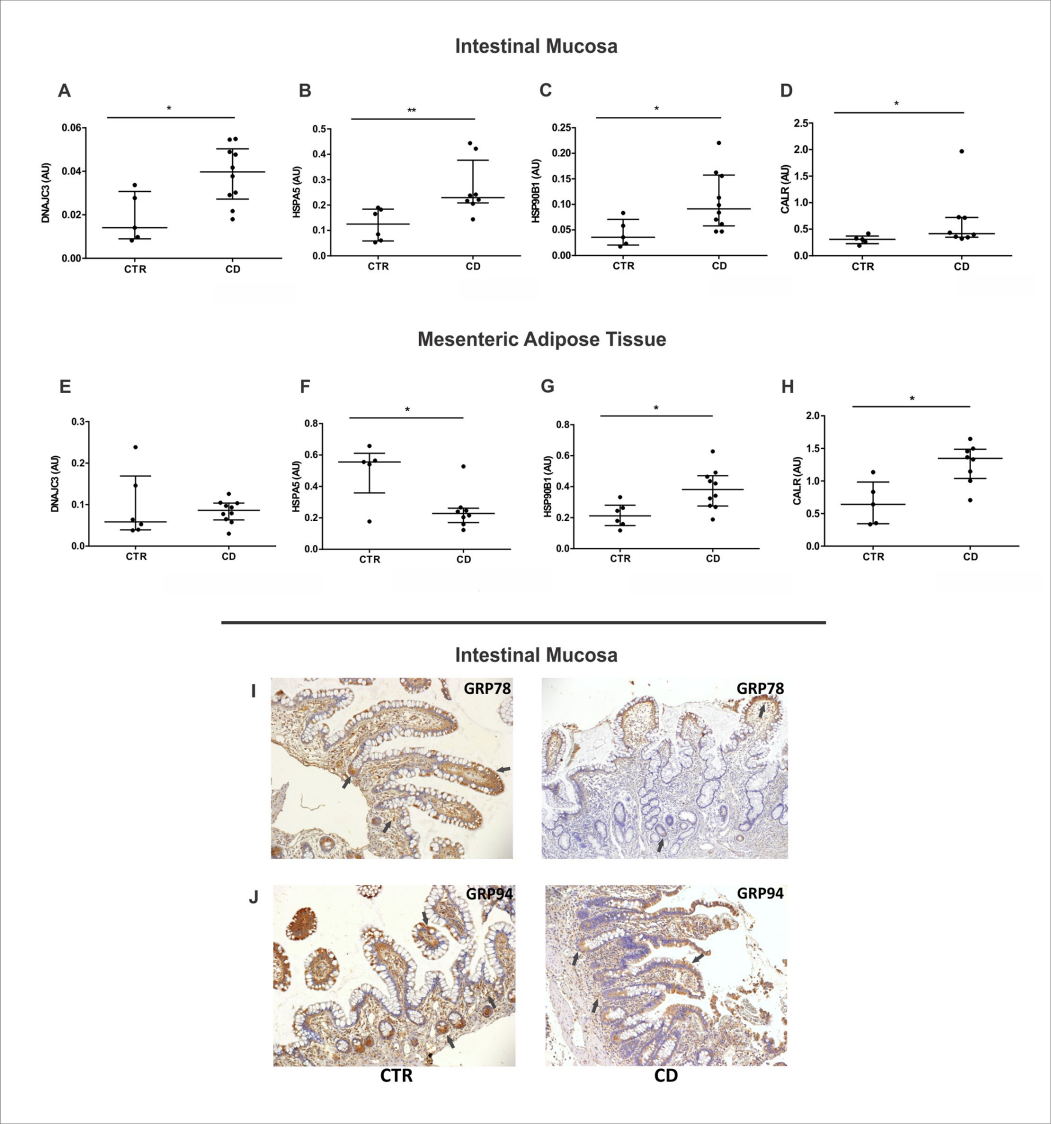
Chaperones and co-chaperone expression in the intestinal mucosa and in the mesenteric adipose tissue (MAT) of Crohn’s disease patients. mRNA levels (qRT-PCR) of *DNAJC3* (A), *HSPA5* (B), *HSP90B1* (C) and *CALR* (D) genes were investigated in the intestinal mucosa of CD patients (CD group) compared to control group (CTR group). mRNA levels of *DNAJC3* (E), *HSPA5* (F), *HSP90B1* (G) and *CALR* (H) genes were also investigated in the MAT of CD and CTR group. *p < 0.05 and **p < 0.01 are considered statistically significant versus control group. Immunohistochemical analysis of GRP78 (I) and GRP94 (J) were performed on paraffin-embedded slides from intestinal mucosa of CD and CTR group. The arrows show labeled epithelial cells by immunohistochemistry. Original magnification 100X. AU: arbitrary unit.

In the last step of this study, we investigated the modulation of chaperones in CD. The activation of UPR branches leads to increased production of chaperones, so this is a broadly used ER stress marker. The intestinal mucosa of CD patients showed an upregulation of the co-chaperone *DNAJC3* (Fig 5A), the *HSPA5* gene that encodes GRP78/BiP protein (Fig 5B), the *HSP90B1* gene that encodes GRP94 (Fig 5C) and the *CALR* gene that encodes the chaperone calreticulin (Fig 5D), corroborating our previous results of ER stress activation in the intestinal mucosa of CD patients. Despite the absence of changes in the co-chaperone *DNAJC3* (Fig 5E), the *HSPA5* gene expression was decreased in the MAT of CD compared to control group (Fig 5F). Interestingly, the transcriptional levels of *HSP90B1* (Fig 5G) and *CALR* (Fig 5H) were increased in MAT of CD patients. Thus, the decrease in *HSPA5* along with increased expression of *HSP90B1* and *CALR* establish a chaperone modulation in the MAT even without ER stress activation. Additionally, we performed immunohistochemistry to investigate the chaperones’ localization in the intestinal mucosa. We observed the GRP78 and the GRP94 immunoreactivities in the apex of the intestinal epithelial villi, in the base of intestinal crypts and in the immune cells from the lamina propria of both control and CD groups (Fig 5I and 5J).

**Fig 6:**
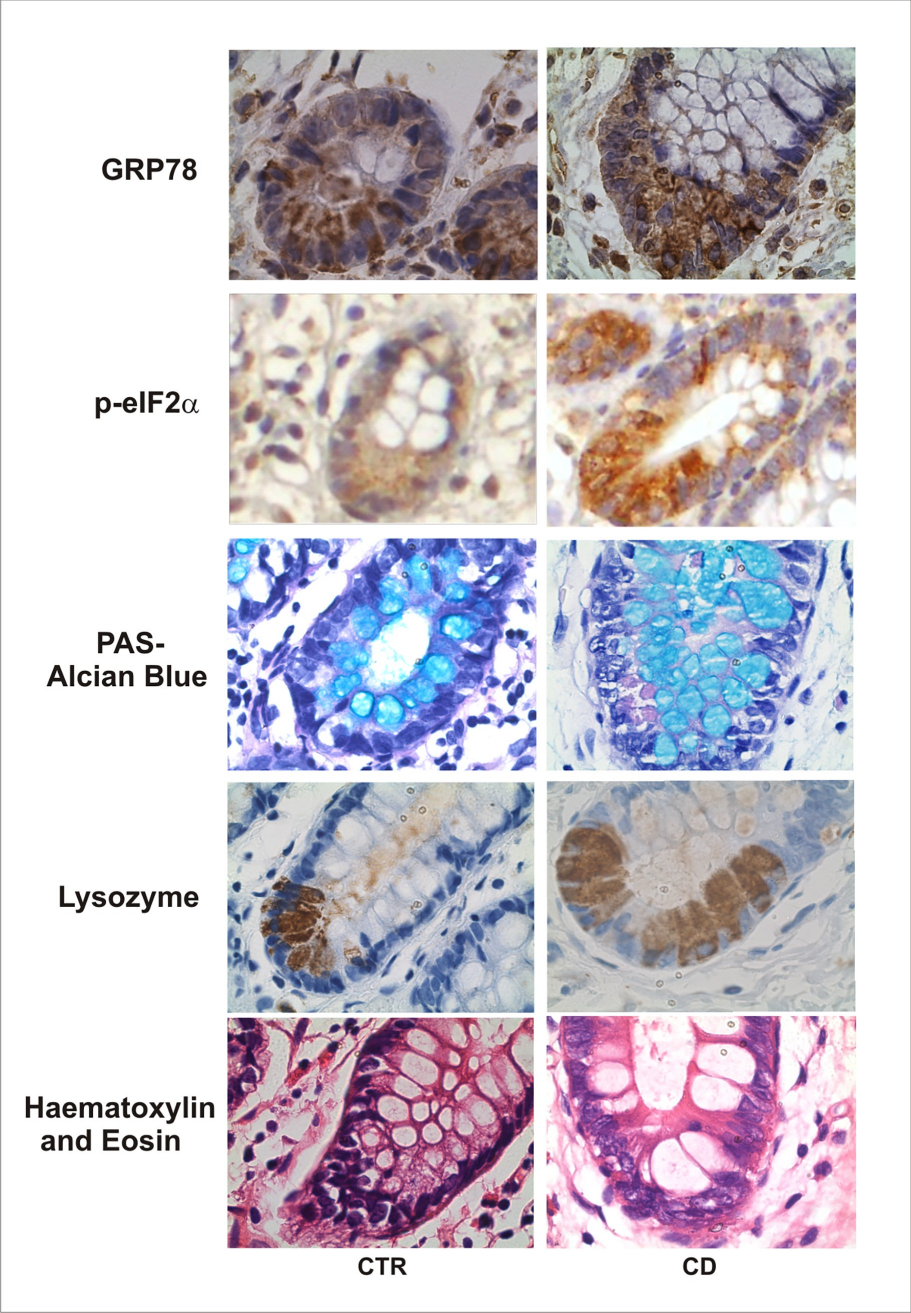
Intestinal crypt characterization of Crohn’s disease (CD) patients and controls. Immunohistochemical analysis of GRP78 and p-eIF2α were performed on paraffin-embedded slides from intestinal mucosa of CD and CTR group. Lysozyme immunohistochemical staining identifies Paneth cells. PAS-Alcian Blue staining reveals cells with mucus. Positive cells for GRP78, p-eIF2-α and lysozyme are shown in brown. Positive cells for PAS-Alcian Blue are shown in light blue. Original magnification 1000X.

To better investigate the ER stress activation in the intestinal crypts, we performed immunohistochemistry for lysozyme and PAS-Alcian Blue, which identify Paneth cells and acidic sulphated mucins respectively. We verified lysozyme, GRP78 and p-eIF2α positive cells at the base of the crypts that are consistent with the localization of Paneth cells in the intestinal epithelium of control and CD groups (Fig 6). For quantitative analysis, we investigate the transcription profile of α-defensin 5 encoded by the gene *DEFA5*. The α-defensins are specifically secreted by Paneth cells localized in the base of the intestinal crypts [23]. Our results showed no differences on *DEFA5* gene expression compared to control group (S3 Fig).

## Discussion

Genetic alterations of ER stress related-genes associate with IBD, specifically in CD and ulcerative colitis (UC) patients [12,24]. Experimental studies point towards ER stress activation as a new pathway driving intestinal inflammation, as demonstrated by *XBP1* deletion in intestinal epithelial cells leading to activation of ER stress and spontaneous enteritis [12,25]. The molecular mechanisms that trigger inflammation in CD differ between the intestinal mucosa and the hypertrophied MAT near the affected intestinal area [3,26]. In the present study, we addressed tissue-specific molecular mechanisms of ER stress in the intestinal mucosa and MAT of CD patients that play a role in CD inflammation.

We initiated the study investigating the three UPR axis activated upon ER stress. The first UPR branch analyzed was IRE1/sXBP1. IRE1α activates c-Jun N-terminal Kinase (JNK)/AP1 pathway and NF-κB, triggering upregulation of inflammatory genes, and contributes to the inflammation and stress-induced cell death [2,14,27]. In fact, our group previously demonstrated increased NF-кB activation in the ileum of CD patients [3]. We observed an increased expression of the *ERN1* transcriptional levels, which induced the spliced form of XBP1 protein in the intestinal mucosa of CD patients. In CD, the epithelial damage of the intestinal mucosa occurs mainly in the apex of the villi, along with increased apoptosis [28], which corroborates our findings of sXBP1 immunoreactivity distribution. Conversely, no differences on ER stress activation were observed in the MAT of CD patients, which suggests the absence of NF-кB activation in this tissue [3].

The second UPR branch evaluated in the intestinal mucosa and in MAT of CD was the ATF6. ATF6 acts in parallel with IRE1 pathway to induce chaperone expression and other elements to increase protein folding, and ERAD components. Our results demonstrate an increased expression of *ATF6* gene in the intestinal mucosa of CD patients, without any concurrent activation in MAT as compared to control group. Interestingly, the increase in *ATF6* transcriptional level does not translate into an augmentation of the activated/cleaved form of ATF6 protein in the intestinal mucosa of CD patients, suggesting no involvement of this UPR axis in CD. The third UPR branch investigated was the PERK/eIF2α pathway. Our results showed increased *EIF2AK3* gene expression, responsible for encoding PERK protein. Additionally, we observed increased p-eIF2α protein content and p-eIF2α/eIF2α ratio in the intestinal mucosa of CD patients, which implies the activation of this UPR axis in CD. Furthermore, we show the p-eIF2α immunoreactivity at the base of the crypts that are consistent with the localization of Paneth cells and at the apex of intestinal villi in both groups. Conversely, no activation occurred in MAT when compared to the control group.

Moreover, in this study we addressed the differences of ER stress-related genes in the intestinal mucosa and MAT of CD patients compared to control group. The expression of the transcriptional regulator *ATF3* occurs early in response to ER stress by p-eIF2α and subsequently ATF4. Along with ATF4, ATF3 engage a transcriptional cascade to initiate apoptosis through activation of CHOP (also known as GADD153 or DDIT3) [29]. Our results show an upregulation of *ATF3* expression in the intestinal mucosa of CD patients, with no differences in MAT as compared to control groups. *STC2* gene, responsible for encoding the secreted glycoprotein stanniocalcin 2, which is induced by ATF4 upon ER stress and plays a role in calcium influx and calcium homeostasis in the cell. Stanniocalcin 2 acts as a pro-survival protein participating in the negative feedback of p-eIF2α and ATF4 [30–32]. Here we show an increase in *STC2* gene expression in the intestinal mucosa of CD patients, with no activation in the MAT compared to control patients.

Another ER stress-related gene investigated in this study was *DNA Damage-Inducible Transcript 3* (*DDIT3*), responsible for encoding the pro-apoptotic CHOP protein. Our results demonstrated an upregulation of *DDIT3* gene expression in the intestinal mucosa of CD patients that suggests an increased cell death in this tissue. Thus, CHOP activation observed in the intestinal mucosa of CD patients could be due to the activation of both UPR branches: IRE1 and PERK/eIF2α. As expected, no differences in CHOP activation were observed in the MAT of CD patients.

Moreover, we investigated the modulation of chaperones and co-chaperone in CD. Initially we evaluated the gene expression of *DNAJC3* that encodes the J-domain co-chaperone DNAJC3. The ER stress induces the expression p58/DNAJC3, which acts as the GRP78 co-chaperone buffering the load of unfolded/misfolded protein in the ER and preventing chronic UPR activation and cell apoptosis [33,34]. Our results demonstrate an increased expression of *DNAJC3* gene in the intestinal mucosa of CD patients, with no activation in MAT compared to control group.

In this study, we also investigated the expression of *HSPA5* gene responsible to encode the GRP78/BiP protein in CD. Upon stress, GRP78/BiP can be reallocated in the cell, where it performs different functions related to signaling, proliferation, apoptosis and inflammation [10,35–37]. An interesting study demonstrates the role of TNFα-induced inflammation in the reallocation of GRP78/BiP to the cytoplasm of epithelial cells in IBD. Cytoplasmic GRP78/BiP interacts directly with IKK complex and activates the NFκB signaling [13]. Here, we show an upregulation of *HSPA5* gene in the intestinal mucosa of CD patients compared to the control group. In addition, the immunohistochemistry analysis showed GRP78 immunoreactivity in the base of intestinal crypts consistent with the localization of Paneth cells. Interestingly, in the MAT of CD patients we demonstrate a decreased expression of *HSPA5* gene compared to the control group. The *HSPA5* downregulation in MAT needs further investigation, but could be due to the activation of STAT1 pathway and increased expression of IL-10 cytokine observed in the MAT of CD patients [3].

Key chaperones in the ER, along with GRP78/BiP, include the heat shock protein GRP94, encoded by *HSP90B1* gene, and the glycoprotein-associated chaperone calreticulin, encoded by *CALR* gene. In our study we demonstrated an upregulation of *HSP90B1* and *CALR* in the intestinal mucosa and MAT of CD patients. The chaperone upregulation is a common feature in UPR response, so this result corroborates with ER stress activation in the intestinal mucosa in CD. Interestingly, in order to maintain the quality control in the ER, a decrease expression or depletion of one chaperone affect the activity and expression of other chaperones. Specifically, the decreased expression of GRP78 is known to induce upregulation of the chaperones GRP94, calreticulin, PDI and Erp57 [38,39]. Thus, the increased expression of the chaperones GRP94 and calreticulin in the MAT of CD patients may be a compensatory mechanism in response to the downregulation of GRP78 observed in this tissue. Further studies are important to understand the regulatory mechanism of upregulation of GRP94 and calreticulin due to decreased GRP78 in the MAT of CD patients.

Therefore, in the present study, we confirmed an increased ER stress activation in the ileal mucosa of CD patients. Bogaert et al. evaluated ileal and colonic samples from colonoscopy examination of CD patients, and they verified ER stress activation only in the colonic mucosa [40]. In our study we used ileal mucosa from surgical specimens of patients who present a severe form of the disease, where the sensibility to detect ER stress activation may be broadly favored. Moreover, we evaluated the ER stress activation and the UPR branches in the MAT of CD patients. Studies of MAT in CD are quite recent and have increasingly attracted the interest of the scientific community mainly due to the discovery that it can be considered a single organ with its own function, physiology and presentation [41,42]. This is an exploratory observational study and the reduced sample size is one of the limitations we encountered, so follow-up studies with a larger cohort are in order to confirm our findings. Another limitation is the restricted age match of the patients: CD disease usually affects young adults, while the diseases or symptoms that lead control individuals to be submitted to a colonoscopy examination or surgery occur later in life.

In summary, for the first time in the literature, we characterized chaperone modulation in MAT of CD patients, with no UPR branch activated. Although this is a descriptive study with a small sample size, conducting research with human subjects brings a contribution to the field that should not be underestimated. Thus, we demonstrated tissue-specific differences in the activation of the pro-inflammatory ER stress mechanism in CD patients, which may be an attractive target for drug development in this field.

## Supporting information

S1 Fig Ponceau-S staining of the Western blot membranes used as loading controls. Ponceau S staining was applied to determine the loading control and protein transfer efficiency in Western blot analysis of sXBP1, ATF6, p-eIF2α and eIF2α expressions in intestinal mucosa as presented in Figs 1, 2 and 3. CD = Crohn’s disease; CTR = control.
(PDF)

S2 Fig Structural histological analysis of intestinal mucosa and mesenteric adipose tissue (MAT) of Crohn’s disease patients. Haematoxylin and Eosin (H&E) staining was performed on paraffin-embedded slides from intestinal mucosa and MAT of Crohn’s disease (CD) patients and controls (CTR); nuclear counterstaining: Mayer’s haematoxylin. Original magnification 200X (A). Immunohistochemical analysis of CD45 was performed on paraffin-embedded slides from intestinal mucosa of CD and CTR groups to show the immune cell infiltration. Original magnification 100X (B).
(PDF)

S3 Fig *DEFA5* gene expression in the intestinal mucosa of Crohn’s disease patients. mRNA levels (qRT-PCR) of DEFA5 gene was investigated in the intestinal mucosa of CD patients (CD group) compared to control group (CTR group). *p < 0.05 and **p < 0.01 are is considered statistically significant versus control group. AU: arbitrary unit.
(PDF)

S1 Table Statistical analyses. The results are reported as median with interquartile range. To check for distributional adequacy, the Kolmogorov-Smirnov test (Chakravart, Laha, and Roy, 1967) was used to investigate if the data follow normal distribution or Gaussian distribution (p>0.1). All data were analyzed using the non-parametric Mann-Whitney Test. In the following table we show the values of U, z-score and p. *p<0.05 and **p<0.01 is the level of significance.
(PDF)

S1 Appendix Electrophoretic gels and blots—Compliance with the digital image. (PDF)
