# Supplementary material for: ER stress activation in the intestinal mucosa but not in mesenteric adipose tissue is associated with inflammation in Crohn’s disease patients
Source: PLoS One. 2019 Sep 26;14(9):e0223105. doi: 10.1371/journal.pone.0223105 (PMC6762147; doi:10.1371/journal.pone.0223105)

S1 Fig. Ponceau-S staining of the Western blot membranes used as loading controls. Ponceau S staining was applied to determine the loading control and protein transfer efficiency in Western blot analysis of sXBP1, ATF6, p-eIF2α and eIF2α expressions in intestinal mucosa as presented in Figures 1, 2 and 3. CD=Crohn's disease; CTR=control.

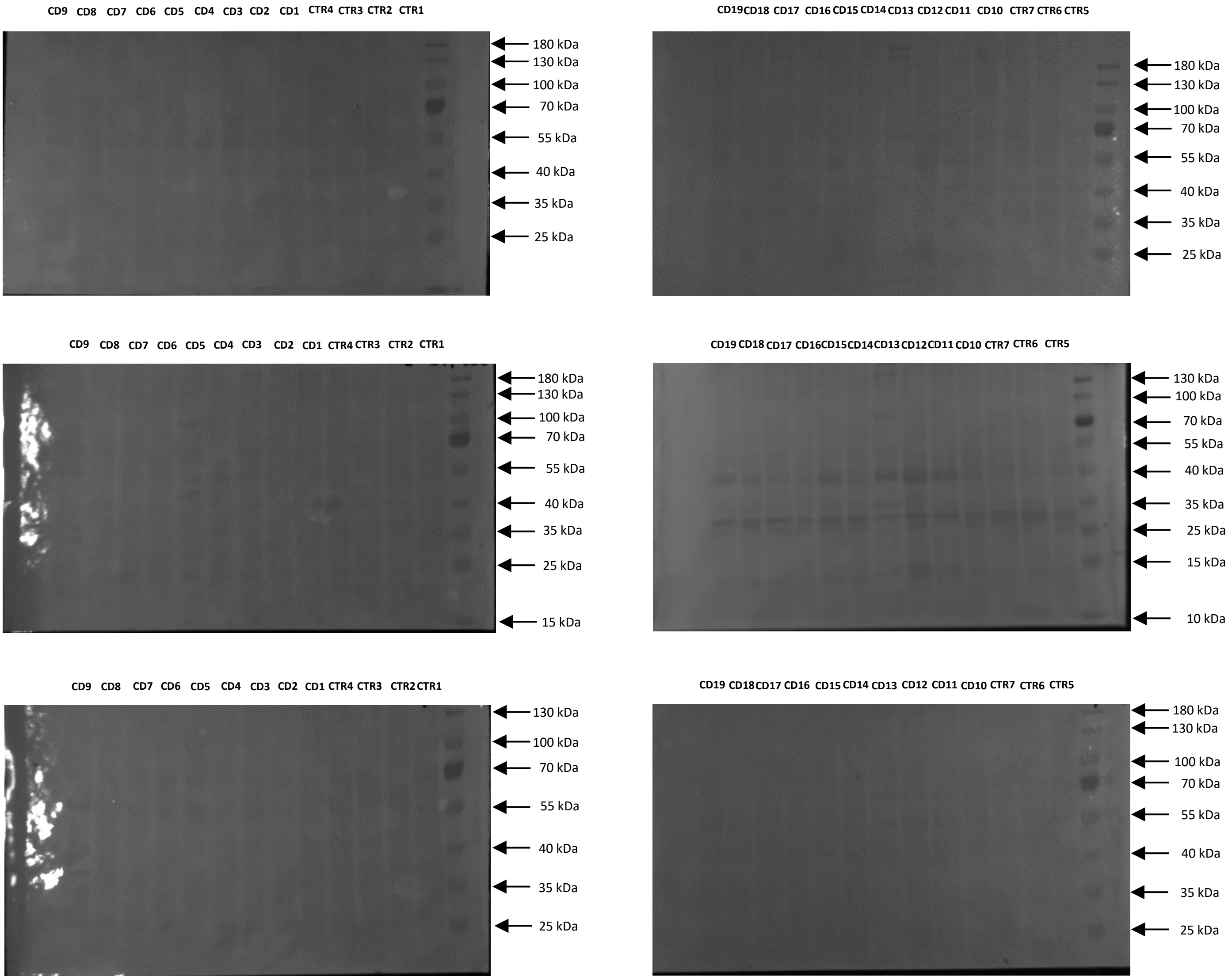

Supplement: S1 Fig — Ponceau S staining was applied to determine the loading control and protein transfer efficiency in Western blot analysis of sXBP1, ATF6, p-eIF2α and eIF2α expressions in intestinal mucosa as presented in Figs 1, 2 and 3. CD = Crohn’s disease; CTR = control. (PDF) [file pone.0223105.s001.pdf]
