# Supplementary material for: ER stress activation in the intestinal mucosa but not in mesenteric adipose tissue is associated with inflammation in Crohn’s disease patients
Source: PLoS One. 2019 Sep 26;14(9):e0223105. doi: 10.1371/journal.pone.0223105 (PMC6762147; doi:10.1371/journal.pone.0223105)

S2 Fig. Structural histological analysis of intestinal mucosa and mesenteric adipose tissue (MAT) of Crohn's disease patients. Haematoxylin and Eosin (H&E) staining was performed on paraffin-embedded slides from intestinal mucosa and MAT of Crohn's disease (CD) patients and controls (CTR); nuclear counterstaining: Mayer's haematoxylin. Original magnification 200X (A). Immunohistochemical analysis of CD45 was performed on paraffin-embedded slides from intestinal mucosa of CD and CTR groups to show the immune cell infiltration. Original magnification 100X (B).

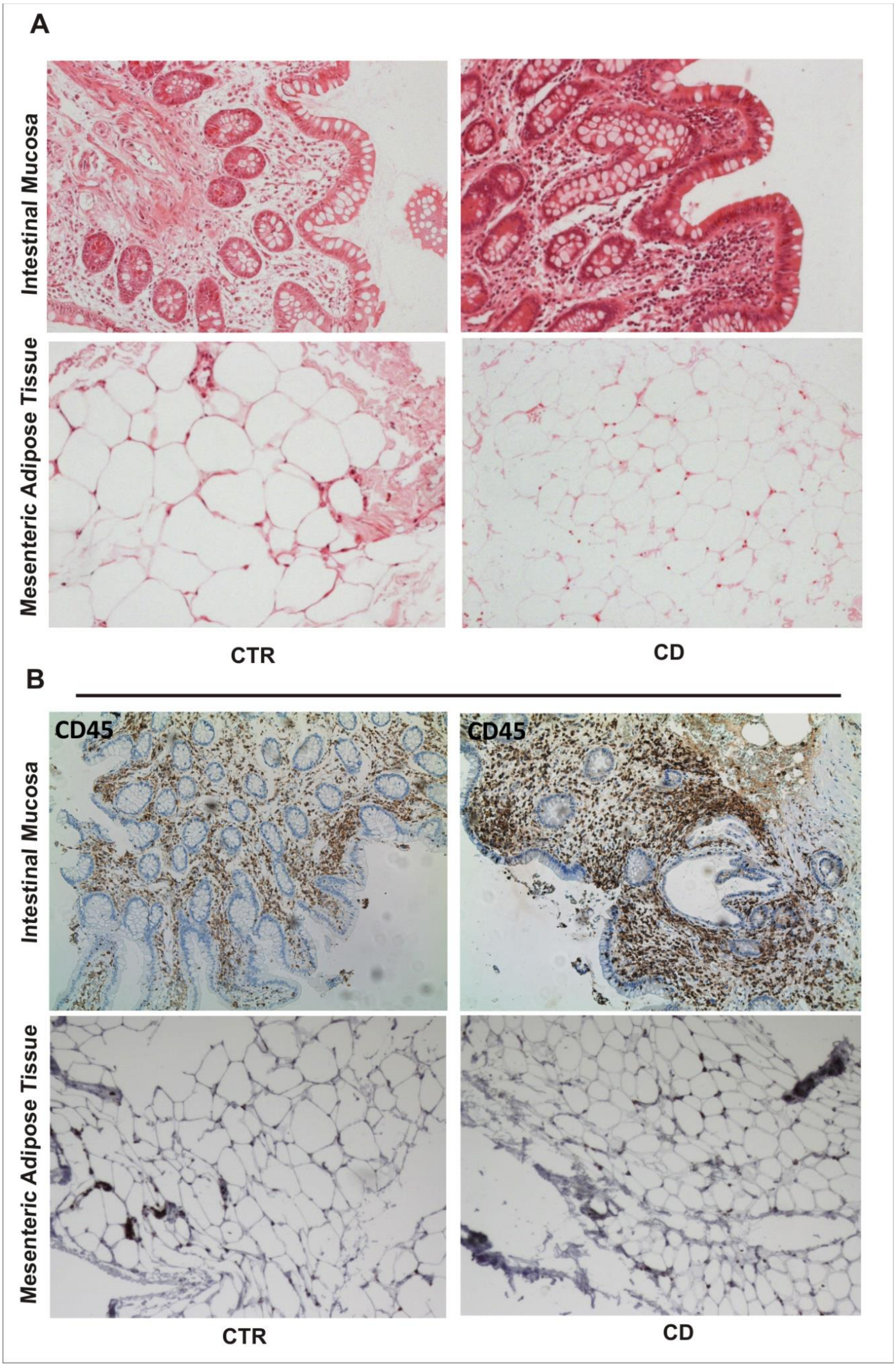

Supplement: S2 Fig — Haematoxylin and Eosin (H&E) staining was performed on paraffin-embedded slides from intestinal mucosa and MAT of Crohn’s disease (CD) patients and controls (CTR); nuclear counterstaining: Mayer’s haematoxylin. Original magnification 200X (A). Immunohistochemical analysis of CD45 was performed on paraffin-embedded slides from intestinal mucosa of CD and CTR groups to show the immune cell infiltration. Original magnification 100X (B). (PDF) [file pone.0223105.s002.pdf]
