# Supplementary material for: ER stress activation in the intestinal mucosa but not in mesenteric adipose tissue is associated with inflammation in Crohn’s disease patients
Source: PLoS One. 2019 Sep 26;14(9):e0223105. doi: 10.1371/journal.pone.0223105 (PMC6762147; doi:10.1371/journal.pone.0223105)

S3 Fig. DEFA5 gene expression in the intestinal mucosa of Crohn's disease patients. mRNA levels (qRT-PCR) of DEFA5 gene was investigated in the intestinal mucosa of CD patients (CD group) compared to control group (CTR group). $*p < 0.05$ and $**p < 0.01$ are considered statistically significant versus control group. AU: arbitrary unit.

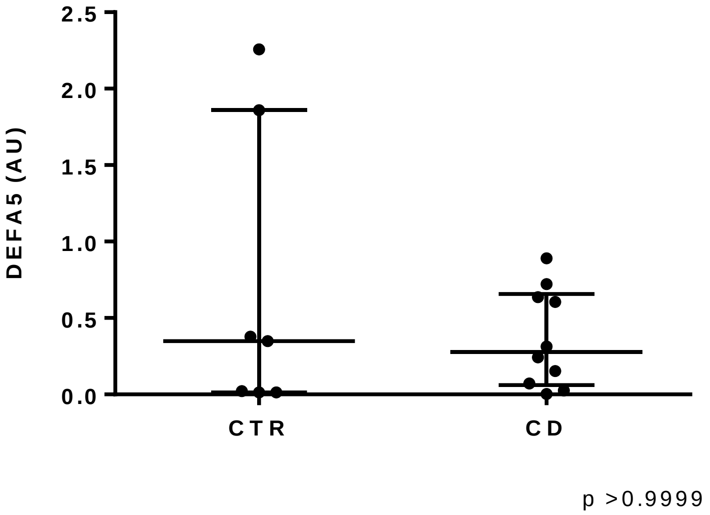

Supplement: S3 Fig — mRNA levels (qRT-PCR) of DEFA5 gene was investigated in the intestinal mucosa of CD patients (CD group) compared to control group (CTR group). *p < 0.05 and **p < 0.01 are is considered statistically significant versus control group. AU: arbitrary unit. (PDF) [file pone.0223105.s003.pdf]
