# Supplementary material for: ER stress activation in the intestinal mucosa but not in mesenteric adipose tissue is associated with inflammation in Crohn’s disease patients
Source: PLoS One. 2019 Sep 26;14(9):e0223105. doi: 10.1371/journal.pone.0223105 (PMC6762147; doi:10.1371/journal.pone.0223105)
Supplement: S1 Table — The results are reported as median with interquartile range. To check for distributional adequacy, the Kolmogorov-Smirnov test (Chakravart, Laha, and Roy, 1967) was used to investigate if the data follow normal distribution or Gaussian distribution (p>0.1). All data were analyzed using the non-parametric Mann-Whitney Test. In the following table we show the values of U, z-score and p. *p<0.05 and **p<0.01 is the level of significance. (PDF) [file pone.0223105.s004.pdf]

S1 Table. Statistical analyses. The results are reported as median with interquartile range. Test for distributional adequacy, the Kolmogorov-Smirnov test (Chakravart, Laha, and Roy, 1967) was used to investigate if the data follow normal distribution or Gaussian distribution ($p>0.1$). All data were analyzed using the non-parametric Mann-Whitney Test. In the following table we show the values of U, z-score and p. *$p<0.05$ and **$p<0.01$ is the level of significance.

Analysis	Tissue	Target	U	z-Score	p-Value
Western Blotting	Ileum	sXBP1	22	-2.54352	0.010975*
		ATF6	40	1.50299	0.132842
		eIF2α	52	0.8093	0.418342
		p-eIF2α	25	-2.15936	0.030823*
		p-eIF2α:eIF2α	24	-2.42791	0.015187*
qPCR	Mesenteric Adipose Tissue	*ATF3*	29	-0.05423	0.95675
		ATF6	25	0.48809	0.625484
		EIF2KA3	28	-0.1627	0.870756
		HSP90B1	6	-2.54893	0.010806*
		HSPA5	6	1.9762	0.048133*
		CALR	3	-2.41535	0.015721*
		STC2	17	-1.35582	0.175159
		DNAJC3	25	-0.48809	0.625484
		ERN1	15	0.6364	0.524519
		DDIT3	19	0.07071	0.943628
	Ileum	*ATF3*	7	-2.68373	0.007281**
		ATF6	5	-2.5338	0.011284*
		EIF2KA3	10	-2.11507	0.034425*
		HSP90B1	6	-2.26578	0.023466*
		HSPA5	3	-2.64654	0.008132**
		CALR	5	-2.12258	0.03379*
		STC2	9	-2.22354	0.02618*
		DNAJC3	6	-2.26578	0.023466*
		ERN1	11	-2.35751	0.018398*
		DDIT3	5	-2.26667	0.023411*
		DEFA5	35	0.0488	0.961083
