# Supplementary material for: ER stress activation in the intestinal mucosa but not in mesenteric adipose tissue is associated with inflammation in Crohn’s disease patients
Source: PLoS One. 2019 Sep 26;14(9):e0223105. doi: 10.1371/journal.pone.0223105 (PMC6762147; doi:10.1371/journal.pone.0223105)
Supplement: S1 Appendix — (PDF) [file pone.0223105.s005.pdf]

S1 Appendix. Electrophoretic gels and blots - Compliance with the digital image.

Figure 1: Activation of IRE1/sXBP1 pathway in the intestinal mucosa and in the mesenteric adipose tissue (MAT) of Crohn's disease patients - Compliance with the digital image. A- Western blot analysis of sXBP1 shown in the article (Figure 2C). **B-** Images of membranes was included. Specific bands of sXBP1 were labeled by a chemiluminescence reaction (SuperSignal West Pico Chemiluminescent Substrate from Pierce Biothecnology, Inc. Rockford, IL), as specified in the study methodology.

A

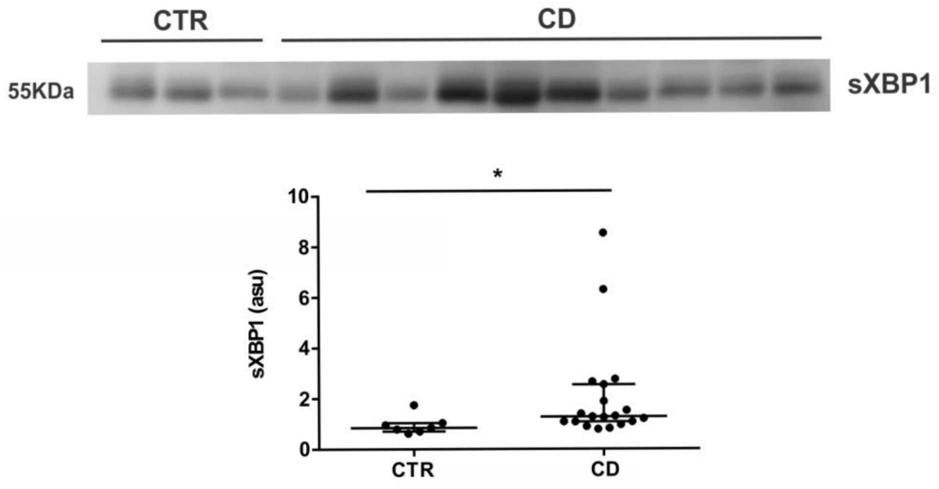

B

sXBP1 - Gel 1

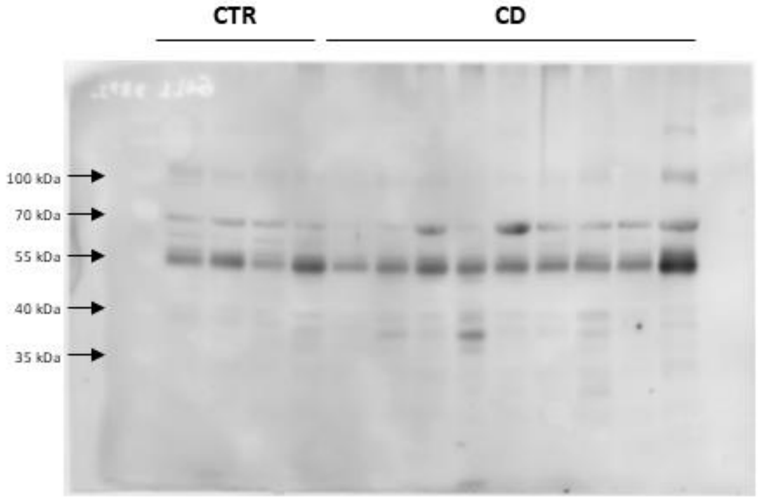

sXBP1 - Gel 2

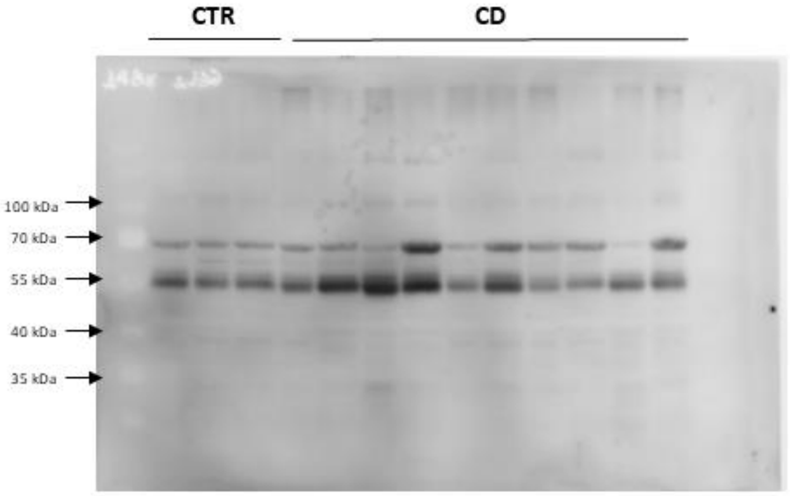

Figure 2: Activation of ATF6 pathway in the intestinal mucosa and in the mesenteric adipose tissue (MAT) of Crohn's disease patients - Compliance with the digital image. A- Western blot analysis of ATF6 shown in the article (Figure 3C). **B-** Images of membranes was included. Specific bands of ATF6 were labeled by a chemiluminescence reaction (SuperSignal West Pico Chemiluminescent Substrate from Pierce Biothecnology, Inc. Rockford, IL), as specified in the study methodology.

A

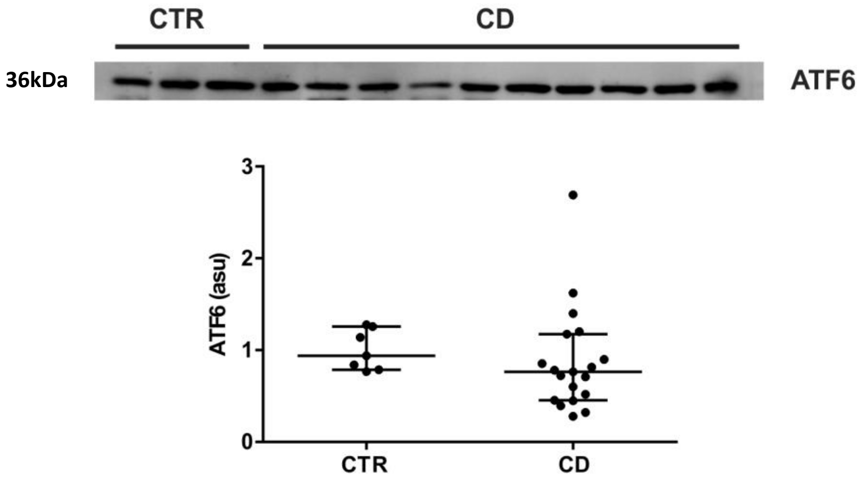

B

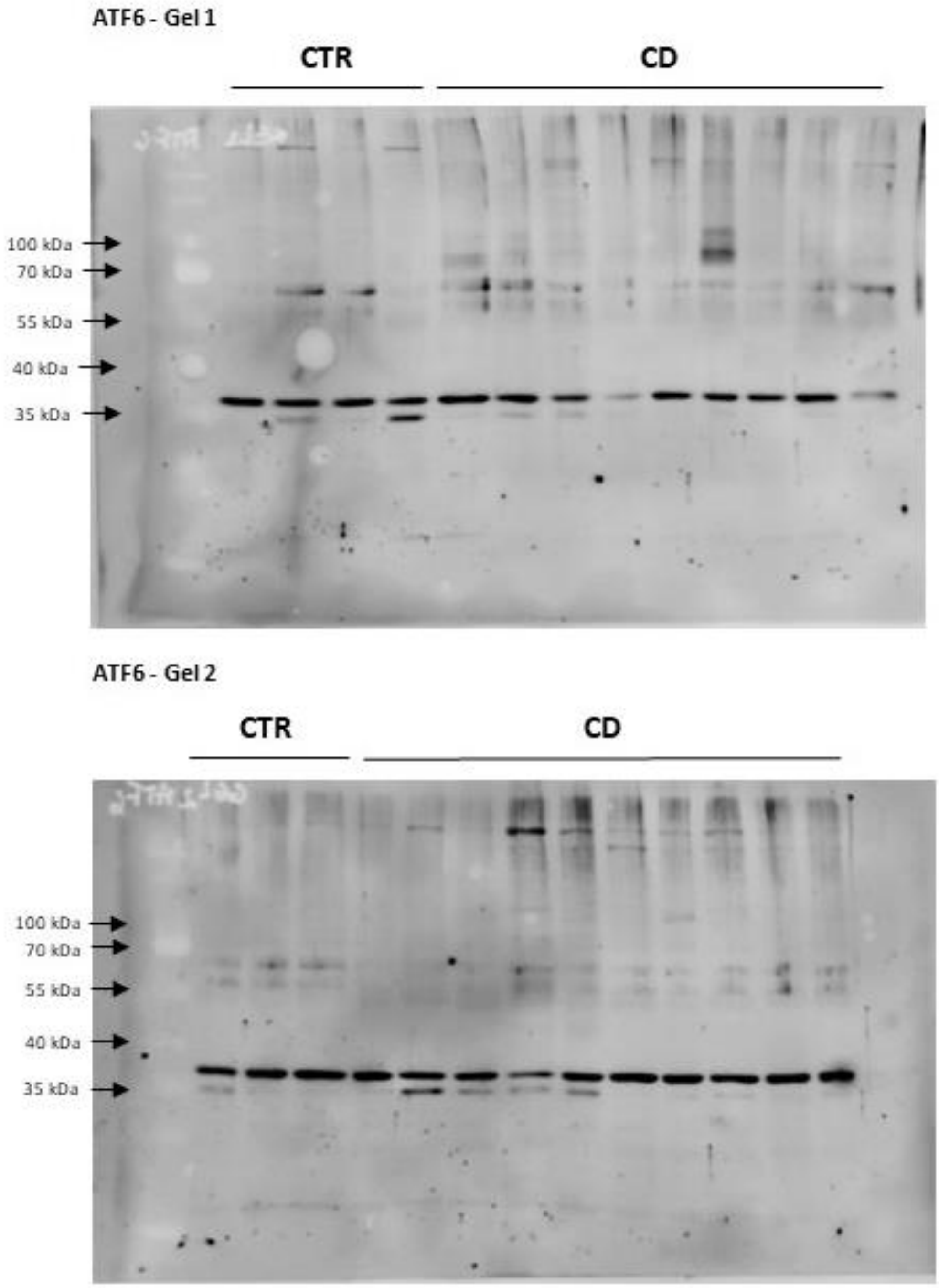
ATF6 - Gel 1
CTR
CD
100 kDa
70 kDa
55 kDa
40 kDa
35 kDa
ATF6 - Gel 2
CTR
CD
100 kDa
70 kDa
55 kDa
40 kDa
35 kDa

Figure 3: Activation of PERK/eIF2α pathway in the intestinal mucosa and in the mesenteric adipose tissue (MAT) of Crohn's disease patients - Compliance with the digital image. A- Western blot analysis of phosphorylated and total form of the protein eIF2α shown in the article (Figure 4C). **B-** Images of membranes was included. Specific bands of p-eIF2α and eIF2α were labeled by a chemiluminescence reaction (SuperSignal West Pico Chemiluminescent Substrate from Pierce Biothecnology, Inc. Rockford, IL), as specified in the study methodology.

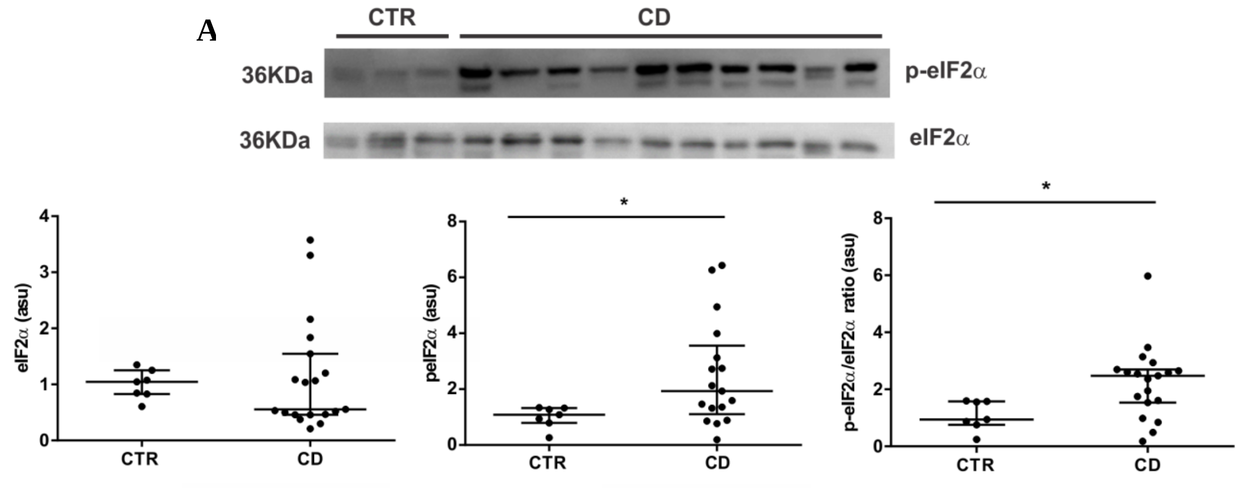

B

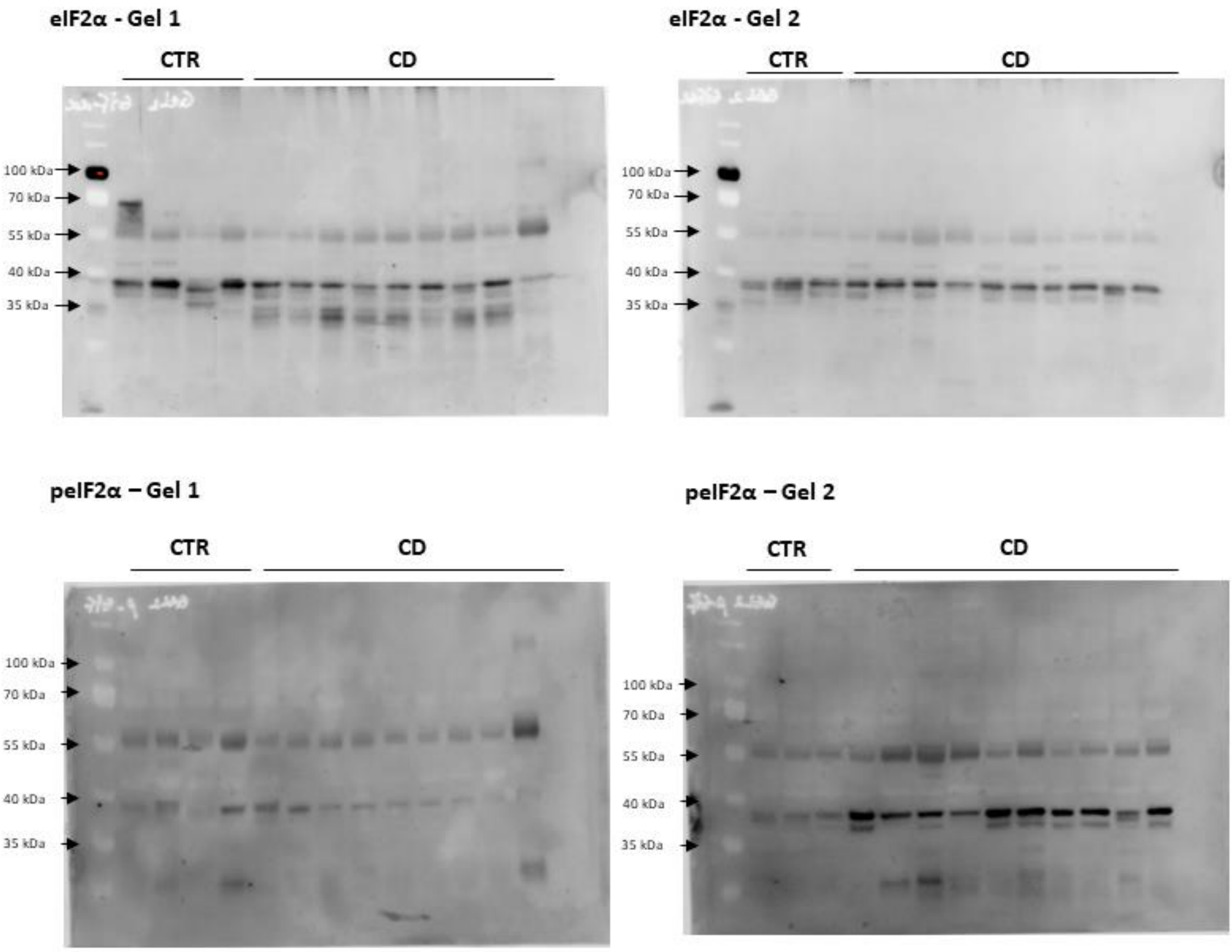
eIF2α - Gel 1
CTR
CD
100 kDa
70 kDa
55 kDa
40 kDa
35 kDa
eIF2α - Gel 2
CTR
CD
100 kDa
70 kDa
55 kDa
40 kDa
35 kDa
peIF2α – Gel 1
CTR
CD
100 kDa
70 kDa
55 kDa
40 kDa
35 kDa
peIF2α – Gel 2
CTR
CD
100 kDa
70 kDa
55 kDa
40 kDa
35 kDa
